# Correlation Analysis between Household Hygiene and Sanitation and Nutritional Status and Female Leprosy in Gresik Regency

**DOI:** 10.1155/2020/4379825

**Published:** 2020-09-30

**Authors:** Flora Ramona Sigit Prakoeswa, Afik Zakie Ilhami, Ratna Luthfia, Aviola Syania Putri, Hardyanto Soebono, Dominicus Husada, Hari Basuki Notobroto, Muhammad Yulianto Listiawan, Anang Endaryanto, Cita Rosita Sigit Prakoeswa

**Affiliations:** ^1^Doctoral Program, Faculty of Medicine, Univesitas Airlangga, Surabaya, Indonesia; ^2^Dermatology and Venereology Department, Faculty of Medicine, Universitas Muhammadiyah Surakarta, Surakarta, Indonesia; ^3^Faculty of Medicine, Universitas Muhammadiyah Surakarta, Surakarta, Indonesia; ^4^Department of Dermatology and Venereology, Faculty of Medicine, Public Health, and Nursing, Universitas Gadjah Mada, Yogyakarta, Indonesia; ^5^Department of Pediatrics, Faculty of Medicine, Universitas Airlangga, Dr. Soetomo General Academic Hospital, Surabaya, Indonesia; ^6^Faculty of Public Health, Universitas Airlangga, Surabaya, Indonesia; ^7^Department of Dermatology and Venereology, Faculty of Medicine, Universitas Airlangga, Dr. Soetomo General Academic Hospital, Surabaya, Indonesia

## Abstract

Leprosy, also known as morbus Hansenʼs disease, is a chronic disease caused by *M. leprae*. Leprosy attacks various parts of the body including nerves and skin. The most important factor in the occurrence of leprosy is the sources of transmission and contact, both from patients and the environment. Household conditions where the person lives and the nutritional status of the individual can be a risk factor for leprosy. Household hygiene and sanitation can be seen from several aspects, like the physical environment of the house, clean water facilities, personal hygiene, availability of latrines, waste disposal facilities, and garbage disposal. This study was aimed to determine the correlation between household hygiene sanitation and nutritional status with females with leprosy in Gresik Regency. This case-control study was conducted in December 2019 in Gresik Regency. The subjects of this study were 74 respondents taken by consecutive sampling techniques. Retrieval of data was carried out using observations from the healthy house component questionnaire, personal hygiene questionnaire, and direct measurement. Data were analyzed using the chi-square test. The results showed significant correlation between physical environment of the house (*p*=0.001, OR = 0.104), clean water facilities (*p*=0.008, OR = 0.261), availability of latrines (*p*=0.018, OR = 0.209), waste disposal facilities (*p*=0.015, OR = 0.291), and personal hygiene (*p*=0.001, OR = 2.850) and female leprosy in Gresik Regency. There is no correlation between nutritional status (*p*=0.085, OR = 0.422) and wastewater disposal waste (*p*=0.183, OR = 0.486) and female leprosy in this study.

## 1. Introduction

Leprosy, also known as morbus Hansenʼs disease, is a chronic disease caused by *M. Leprae*. Leprosy affects various parts of the body including nerves and skin. Skin lesions are signs that can be observed from the outside. If it is left untreated, leprosy can become progressive, causing damage to the skin, nerves, limbs, and eyes [1].

Leprosy is generally found in developing countries as a result of the countryʼs limited ability to provide adequate services in the fields of health, education, and socioeconomic welfare to the community. In 2011, 83% of new cases were detected worldwide comprising 58% cases in India, 16% cases in Brazil, and 9% cases in Indonesia [2].

In 2000, leprosy elimination status in Indonesia showed no progression. This can be seen from the number of new leprosy case findings in more than twelve years ranging from six to eight per 100,000 population, and the prevalence is between eight and ten per 100,000 population annually. The presence of a new leprosy case means that there is a failure in the transmission termination of *M. leprae* [3].

Leprosy is found in 14 provinces of Indonesia, with the prevalence of 1 case per 10,000 population [4]. Almost all eastern-part provinces of Indonesia are areas with high leprosy burden, while East Java province is the only province in the western part of Indonesia with a high number of leprosy [1].

The number of people affected by leprosy in East Java is 4807 people, making East Java the province with the highest leprosy cases in Indonesia. The number of leprosy patients in East Java from 2015 to 2017 decreased from 4013 people to 3373 people [4]. In Gresik Regency, from 2010 to 2017, there were 104–150 leprosy patients, and there were eight districts which have high leprosy cases in Gresik Regency, namely, Wiringanom, Tambak, Pancen, Ujung pacing, Bungah, Sidayu, Dukun, and Kedamean. The leprosy prevalence is 1.24 out of every 10,000 population, and about 5–7% children were affected by leprosy, while those with second-level physical disability are 12.38% [5].

Sex distribution of leprosy patients shows that males are more affected than females [4]. However, previous study in Indonesia reported that females with leprosy experienced problems of discrimination and stigmatization related to marriage more than men [6]. Women's health, especially those in childbearing age, can affect the immunity dysregulation of her children, if a woman during her pregnancy experienced infection, malnutrition, obesity, and exposure to cigarette smoke [7]. Failure to resolve the causes of immune system in communities that live in leprosy endemic environments makes transmission of *M. leprae* bacteria easier since the host becomes more susceptible to leprosy [8]. The dominant role of women in taking care of their family increases the chance of female with leprosy to transmit the disease to other house members, especially their children. Moreover, females in developing countries tend to get late health treatment in health care for any health-related problems. [9].

Paredes and Morales [10] reported that poor housing in dense and unstructured residential can increase the risk of leprosy. Contact history is related to the incidence of leprosy. People with 4–10 years of contact with leprosy patients have a greater risk than those who have less than 4 years of contact with patients. This concept is supported by the tendency of leprosy to occur more in people who are in the same household contact than those who are not in the same household [11].

The two most important factors in the occurrence of leprosy are sources of transmission and contact, from both patients and from the environment. The physical environment of the house is part of the physical environment that can affect the health of individuals and society. House, which is a place to live in, must meet health requirements such as good ventilation and good humidity, residential density in accordance with the area of the house, and nonsoil floor. Termination of leprosy transmission chain with appropriate interventions can be done if the infection process of leprosy transmission can be known [4].

Previous study reported that there was no correlation between clean water and the incidence of leprosy. The clean water source among leprosy patients did not contain acid-resistant bacteria which is a parameter of leprosy bacteria [12]. However, another study showed different results; there was a correlation between the source of clean water and the incidence of leprosy in Konang, Geger, and Bangkalan districts [13].

The environment is also a reproduction place for various bacteria, including leprosy bacteria. The house is part of the physical environment that can affect the health of individuals and society. Houses used as residence should meet health requirements such as having wastewater disposal, garbage disposal, and the availability of healthy latrines [14].

Based on previous study, there was no correlation between wastewater disposal facilities (OR = 2.500) and waste disposal (OR = 0.625) and the incidence of leprosy (*p* value > 0.05). Hence, wastewater disposal and waste disposal are not a risk factor for leprosy [14].

According to Ratnawati [14], latrine is a risk factor that has a significant correlation with the leprosy transmission (*p* value < 0.05) [14]. Latrines which do not fulfill requirements have 5.179 times greater chance to transmit leprosy than qualified latrines. Based on the Health Minister Decree Number 829 of 1999 concerning Housing Health Requirements, latrines that meet health requirements are goose-necked latrines with septic tanks [14].

Personal hygiene is closely related to community hygiene and has mutual influence. The more people who pay attention to the maintenance and improvement of their health, the better the health of the community. Poor personal hygiene is a reflection of environmental conditions and the unhealthy behavior of individuals. Leprosy can be prevented through improvement in personal hygiene [15]. Some studies indicate that individual hygiene factors can influence the transmission of leprosy. Research conducted by Aning et al. shows that there is a correlation between individual hygiene and the incidence of leprosy in the Tanjung Area primary health care [15]. Meanwhile, a study conducted by Susanti and Azam found that individual hygiene was not related to the occurrence of leprosy (*p*=0.077) [16].

Leprosy affects many people with low socioeconomic status. This is associated with low endurance, poor nutrition, and poor environment and hygiene [17]. Based on a study conducted by Zuhdan et al. [17], poor nutritional factors affect leprosy (*p*=0.001; OR = 5.04; and 95% CI = 2.761–9.182).

For the practice aspect, this can be an effort to reduce the number of new leprosy cases in Gresik Regency by paying more attention to the promotive and preventive aspects in the effort to break the chain of leprosy transmission in the environmental health status.

The benefit of this research in theoretical aspects is to get information about the correlation between physical environment of the house and clean water facilities with female leprosy in Gresik Regency. The purpose of this study was to determine the correlation of hygiene conditions and sanitation of the home environment and nutritional status with female leprosy in Gresik Regency. The hypothesis of this study was as follows: there is a correlation between the physical environment of the house, clean water facilities, personal hygiene of occupants of the house, availability of latrines, wastewater disposal facilities, waste disposal facilities, and nutritional status and female leprosy in Gresik Regency.

Based on the literature, there has been no study examining the correlation between hygiene, sanitation, home environment, and nutritional status and females with leprosy in Gresik Regency before. Considering the effect given of the incidence on female leprosy, we aimed to know the correlation between household hygiene, sanitation, and nutrition and incidence of female leprosy in Gresik regency to be used as information to stop leprosy transmission in endemic areas using promotive and preventive approach. This research is not the first study in Gresik Regency, but in previous studies, the focus of the research lies in the field of sociology [5].

## 2. Methods

The study samples were females with leprosy who met the inclusion criteria. The sampling method used was consecutive sampling. Case control study design was performed. The tools used were personal data questionnaires, measuring meters, and healthy home assessment forms taken from the Ministry of Health and revalidated to assess the house environment consisting of the physical environment of the house, clean water facilities, availability of latrines, wastewater disposal facilities, and waste disposal facilities. Individual hygiene data collection was carried out by interview using questionnaire, and nutritional status data were obtained by using an observation sheet of BMI measurements. The measurements used are weight measuring devices under the Camry brand and the microtoise height measuring device under the Gea Medical brand.

The location of this study was in Gresik Regency. Data collection was performed after respondents received an explanation before signing the informed consent. The activity was continued by filling out the questionnaire, observing, and measuring directly in the field. The type of data collected was divided into two, namely, primary data (obtained through observation and interviews with respondents) and secondary data (obtained through data from the Gresik District Health Office to determine the type of leprosy).

The inclusion criteria used in this study were (1) productive age/childbearing females aged 20–49 years old, (2) diagnosed with leprosy, and (3) agreed to participate and sign the informed consent in this study. The physical environment of the house was seen directly from its condition; the interpretations were a good house (≥4) and a bad house (<4). Then, for the clean water facility variable, the questions were asked about the source of the clean water facility; the interpretations were clean water (≥3) and unclean water (<3). The availability of latrines was seen directly in its condition, with good latrine interpretation (≥3) and bad latrines (≤3), while the interpretations of good waste disposal variables (≥3) and bad waste disposal (≤3) were as follows: the interpretations of good garbage disposal variables were good garbage disposal (≥3) and bad waste disposal (≤3). Nutritional status variables were interpreted according to existing IMT standards. This study was approved by the Health Research Ethics Commission Dr. Soetomo Surabaya.

This study was analyzed using the SPSS-24.0 application. The analysis of the effect of independent and dependent variables used bivariate and multivariate analyses. Bivariate analysis was used to determine the level of significance of the effect of independent variables with the dependent variable.

## 3. Results

This study was conducted in December 2019 in Gresik Regency. Respondents who participated in this study amounted to 74 respondents who met the inclusion criteria. The data of this study were taken directly and the questionnaire was filled directly by the research subjects, namely, females affected by leprosy in Gresik Regency; the characteristics of the subjects of this study can be seen in Table 1.

There were 74 subjects consisted of 37 female leprosy patients and 37 healthy women, and 54 (73.0%) of them had good physical environment while 20 (27.0%) others had bad physical environment. Subjects who had good clean and poor water facilities were 36 (48.6%) and 38 (51.4%), respectively. Good latrines were owned by 63 subjects (85.1%) and the other 11 subjects (14.9%) had bad latrines. The number of subjects with and without leprosy was similar, 37 each. Most subjects (78.4%) had bad wastewater facility. Waste disposal was mostly bad (69.9%) (Table 1).

The correlation test of house physical environment with leprosy on females obtained *p* value of <0.001 and the OR value of 0.104, showing that females with poor physical environment have a risk of 0.104 to be affected by leprosy (Table 2).

In the correlation of clean water facilities with leprosy females, the *p* value was 0.008 and OR value was 0.261, indicating that females with poor clean water facilities had a risk of 0.261 times to get leprosy. In relation to the availability of latrines and females with leprosy, we obtained the value of *p*=0.018 and OR value of 0.209, indicating that females with poor latrines had a risk of 0.209 times to get leprosy. The correlation test between wastewater disposal facilities and females with leprosy showed the *p* value of 0.183 and OR of 0.486, indicating no correlation between the two. In the correlation between waste disposal facilities and females with leprosy, the *p* value of 0.015 and OR of 0.291 indicated that females with poor waste disposal facilities had a risk of 0.291 times to suffer leprosy. Personal hygiene was found to be related to female leprosy (*p* < 0.001 and OR = 2.850). Therefore, females with poor personal hygiene had a risk of 0.291 times to be affected by leprosy. Nutritional status was found to be not correlated with female leprosy (*p*=0.085 and OR = 0.422).

The logistic regression test obtained house physical environment *p* value = 0.002, waste disposal facilities *p* value = 0.035, and personal hygiene *p* value = 0.001. The OR value exponentiated estimate (exp. *B*) of the house physical environment variable was 0.030 so that subjects with a bad physical environment would have a risk of 0.030 times to be infected by leprosy. The OR value (exp. *B*) of the garbage disposal facility variable was 0.848, meaning that subjects with a poor physical environment would have a 0.848-fold risk of leprosy. The OR value (exp. *B*) of the individual hygiene variable is <0.001; thus, subjects with a poor physical environment would have a risk of <0.001 times for leprosy (Table 3).

## 4. Discussion

Table 2 shows the results of the bivariate analysis test using the chi-square test between household hygiene and sanitation and females with leprosy in Gresik Regency. The results of the bivariate analysis between house physical environment and females with leprosy obtained *p* value <0.001, because when the value of *p* < 0.05, then there is a significant correlation between house physical environment and females with leprosy. In this study, the houseʼs physical environment was assessed starting from the ceiling, walls, floors, and ventilation.

This result is consistent with previous study which stated that there was a significant relationship between the physical condition of the house and the leprosy incidence. Many houses in Bandar Lampung city do not meet the criteria for healthy ventilation. Health ventilation area needs to be more than 10 percent of the floor area [12]. *M. leprae* is found in dust and water. Therefore, the physical condition of the house that meets health requirements is needed to prevent the spread of *M. leprae* bacteria in the environment. The physical condition of the house includes the types of building materials such as the types of walls, floors, and roofs and the location of the house. The type of home building material will affect the water infiltration and the amount of dust in the house.

Various types of diseases can arise due to a bad environment. A healthy house will provide good health to people living there. If the floor of the house is made of nonwaterproof material, it can cause water to seep into the house, turning the environment to become unhealthy. *M. leprae* bacteria tend to live in places with unhealthy environment. Based on our observations, the floors of the houses of the subjects affected by leprosy are mostly made of soil. Thus, they can be an ideal place for the growth of *M. leprae* bacteria. House floors that are not waterproof will absorb water from the soil, thereby increasing humidity, and can act as a reservoir for *Mycobacterium leprae* [18].

The results of bivariate analysis between clean water facilities and females with leprosy obtained a *p* value of 0.008, because when the value of *p* < 0.05, then there is a correlation between clean water facilities and female leprosy. This result is similar with previous research conducted by Nurcahyati et al. [13] reporting the distribution of new leprosy cases based on environmental and socioeconomic factors in the districts of Konang, Geger, and Bangkalan, which showed the relationship between water sources and the incidence of new leprosy (*p* < 0.001) [13]. The clean water facility is one of the environmental factors that is suspected to be a source of transmission in endemic areas, as evidenced by a large number of new cases in endemic areas where there is no clear history of contact with leprosy patients. This is similar to our observations where several areas in Gresik Regency still use their water sources from ponds for bathing and other needs.

We found that the availability of latrines is significantly related to leprosy females. This finding is in line with the research conducted by Ratnawati [14] which stated that healthy latrines had a significant relationship in the incidence of leprosy (*p*=0.045, OR = 5.179) [14]. The chance of people who live in a house with unhealthy toilets to be infected by leprosy is 5.179 times greater than those with healthy latrines. Based on the Health Minister Decree No. 829 of 1999 concerning Housing Health Requirements, latrines that meet health requirements are goose-necked latrines with septic tanks [14].

The results of bivariate analysis between wastewater disposal facilities and females with leprosy obtained a *p* value of 0.183, so that it can be inferred that wastewater disposal is not significantly correlated with leprosy. Other study also reported similar finding that wastewater disposal is not a risk factor [14].

In our study, the chi-square test obtained a significant relationship between waste disposal facilities and females with leprosy. A study conducted in Brazil found that waste disposal facilities were associated with leprosy (95% CI: 1.91–27.98). Good waste disposal means that there is garbage collection and there is no littering of waste scattered or managed carelessly. Waste management by burning or stockpiling can increase the risk of leprosy by 7.3 times greater than the proper and correct waste management [19].

The results of bivariate analysis between individual hygiene and females with leprosy obtained a significant relationship between personal hygiene and females with leprosy. A study conducted by Susanti and Azam [16] also found that personal hygiene was associated with leprosy (*p*=0.007) [16]. Tarmisi et al. [20] also reported similar finding that personal hygiene is a risk factor for the occurrence of leprosy [20]. Rarely using soap when washing hands before handling food and eating or after carrying out other activities is also one of the causes of leprosy germs' easy infection. By using soap, the dirt can be cleaned, and germs can be killed; without using soap, dirt and germs are still left in the hand.

Prevention of leprosy can be done by increasing personal hygiene, including skin care, hair maintenance, and nails. Because leprosy transmission is very much influenced by direct contact with skin and hair follicles, they need to be kept clean.

The results of our bivariate analysis demonstrated that nutritional status and leprosy of females were not correlated. In this study, the nutritional status termed “under” was nutritional status below BMI. A study conducted in India suggested that one of the risk factors worsening the condition of leprosy is overnutrition, a condition in which the nutritional status of patients is categorized overweight or obese on a BMI scale [21].

Our logistic regression tests on house physical environment found that females with bad houses physical environment had a risk of 0.030 times to be infected by leprosy, while poor physical environment had a 0.848-fold risk for causing leprosy.

The limitation of this study is that the house environment observation in this study used the case-control method, so that the authors did not know whether the physical environment of the house has been renovated before or not, making the results less optimal.

## 5. Conclusions and Suggestions

There is correlation between house physical environment, clean water facilities, availability of latrines, waste disposal facilities, and personal hygiene in females leprosy in Gresik Regency, and there is no correlation between wastewater disposal facilities and nutritional status in females with leprosy in Gresik Regency from bivariate analysis test. The logistic regression test shows there is correlation between house physical environment (*p*=0.002), waste disposal facilities (*p*=0.035), and personal hygiene (*p*=0.001).

Awareness and action to maintain sanitation hygiene at home and to maintain nutritional status are needed in our community in order to avoid the case of leprosy of females in particular. The maintenance of sanitation hygiene can be done by maintaining the condition of the physical environment, clean water facilities, availability of latrines, waste disposal facilities, and personal hygiene. We are looking forward to other in-depth studies using larger samples related to the household hygiene sanitation problems with female leprosy on different subjects and research designs such as cohort studies so that the results obtained from similar studies are more perfect.

## Figures and Tables

**Table 1:** Characteristics of research subjects.

Variable	Amount	Percentage (%)
Home physical environment		
Good	54	73
Bad	20	27

Water facilities		
Good	36	48.6
Bad	38	51.4

Toilet availability		
Good	63	85.1
Bad	11	14.9

Wastewater facility		
Good	16	21.6
Bad	58	78.4

Waste disposal		
Good	23	31.1
Bad	51	69.9

Personal hygiene		
Good	57	77
Bad	17	23

Nutritional status		
Good	49	66.2
Bad	25	33.8

Leprosy		
Have	37	50
Do not have	37	50

**Table 2:** Relationship between household hygiene and sanitation with leprosy.

			Leprosy			*p* value	OR value
			Having leprosy	Not having leprosy	Total		
House physical environment	Bad	*N*	17	3	20	0.001	0.104
		%	85.0	15.0	100		
	Good	*N*	20	34	54		
		%	37.0	63.0	100		
	Total	*N*	37	37	74		
		%	50.0	50.0	100		

Clean water facilities	Bad	*N*	19	8	27	0.008	0.261
		%	70.4	29.6	100		
	Good	*N*	18	29	47		
		%	38.3	61.7	100		
	Total	*N*	37	37	74		
		%	50	50	100		

Latrines' availability	Bad	*N*	11	3	14	0.018	0.209
		%	78.6	21.4	100		
	Good	*N*	26	34	60		
		%	43.3	56.7	100		
	Total	*N*	37	37	74		
		%	50	50	100		

Wastewater disposal facilities	Bad	*N*	30	25	55	0.183	0.486
		%	54.5	45.5	100		
	Good	*N*	7	12	19		
		%	36.8	63.2	100		
	Total	*N*	37	37	74		
		%	50	50	100		

Waste disposal	Bad	*N*	29	19	48	0.015	0.291
		%	60.4	39.6	100		
	Good	*N*	8	18	26		
		%	30.8	69.2	100		
	Total	*N*	37	37	74		
		%	50	50	100		

Personal hygiene	Bad	*N*	17	0	27	0.001	2.850
		%	100.0	0.0	100		
	Good	*N*	20	37	47		
		%	35.1	64.9	100		
	Total	*N*	37	37	74		
		%	50	50	100		

Nutritional status	Bad	*N*	16	9	27	0.085	0.422
		%	64.0	36.0	100		
	Good	*N*	21	28	47		
		%	42.9	57.1	100		
	Total	*N*	37	37	74		
		%	50	50	100		

**Table 3:** Logistic regression test.

Independent variable	*B*	OR exp. (*B*)	95% CI for exp. (*B*)	Sig.
Physical environment house	−3.519	0.030	0.003–0.284	0.002
Clean water facilities	−0.606	0.112	0.112–2.668	0.454
Latrines' availability	−0.441	8.561	0.048–8.561	0.738
Waste disposal facilities	−2.342	0.848	0.011–0.848	0.035
Personal hygiene	−21.819	0.000	0.001	0.001
Constant	47.171	3.062	—	0.998

Exp. (B) refers to exponentiated estimate.

## Data Availability

The questionnaire data used to support the findings of this study are included within the supplementary information files.

## Supplementary Materials

Supplementary Materials The questionnaire data used to support the findings of this study are included within the supplementary information files.

## References

[1] EDCD (2018). *National Guidelines on Leprosy Control Program*.

[2] Oliveira M. B. B. d., Diniz L. M. (2016). Leprosy among children under 15 years of age: literature review. *Anais Brasileiros de Dermatologia*.

[3] WHO (2015). *Global Leprosy Update, 2015: Time for Action, Accountability and Inclusion*.

[4] Indonesian Ministry of Health Indonesia Health Profile 2018. * Indonesian Ministry of Health*.

[5] Shofiyah A. B. T., Fahimah S. (2017). Leprosy and its problems: sociology study in banyuurip village ujungpangkah Gresik. *Media Komunikasi Sosial Keagamaan*.

[6] van Brakel W. H., Sihombing B., Djarir H. (2012). Disability in people affected by leprosy: the role of impairment, activity, social participation, stigma and discrimination. *Global Health Action*.

[7] PrabhuDas M., Bonney E., Caron K. (2015). Immune mechanisms at the maternal-fetal interface: perspectives and challenges. *Nature Immunology*.

[8] Sadhu S., Mitra D. K. (2018). Emerging concepts of adaptive immunity in leprosy. *Frontiers in Immunology*.

[9] Sarkar R., Pradhan S. (2016). Leprosy and women. *International Journal of Womens’ Dermatology*.

[10] Franco-Paredes C., Rodriguez-Morales A. J. (2016). Unsolved matters in leprosy: a descriptive review and call for further research. *Annals of Clinical Microbiology and Antimicrobials*.

[11] Ratnawati R., Rahfiludin M. Z., Kartasurya (2018). Relationship between home physical and non-physical environment with anti phenolic glicolipid-1 IgM antibody levels in children of leprosy patients (house physical and non-environment associated with levels of IgM anti phenolic glicolipid-1 (PGL-1). *Period Skin Heal and Gender*.

[12] Wicaksono M., Faisya A., Budi I. (2015). Physical environment home and characteristics of respondents related to the disease of leprosy clinical in the city of bandar lampung. *Jurnal Ilmu Kesehatan Masyarakat*.

[13] Nurcahyati S., Wibowo A. (2016). Distribution of new leprosy cases based on environmental and social economic factors in Konang and geger subdistrict, bangkalan regency. *Jurnal Wiyata*.

[14] Ratnawati R. (2016). Factors related to the risk of leprosy (Morbus hansen). *Tunas-Tunas Ris Kesehatan*.

[15] Aning H. N., Haidah N., Nerawati A. D. (2018). Relationship of individual characteristics with leprosy. *Gema Kesehatan Lingkungan*.

[16] Susanti K. N., Azam M. (2016). Hubungan status vaksinasi bcg, riwayat kontak dan personal hygiene dengan kusta di kota pekalongan. *Unnes Journal of Public Health*.

[17] Zuhdan E., Kabulrachman K., Hadisaputro S. (2017). Faktor-faktor yang mempengaruhi kejadian kusta pasca kemoprofilaksis (studi pada kontak penderita kusta di Kabupaten sampang). *Jurnal Epidemiologi Kesehatan Komunitas*.

[18] Siswanti S., Wijayanti Y. (2018). Faktor risiko lingkungan kejadian kusta. *Journal of Public Health Research and Development*.

[19] Rodrigues T. S. V., Gomes L. C., Cortela D. C. B., Silva E. A., Silva C. A. L., Ferreira S. M. B. (2019). Factors associated with leprosy in children contacts of notified adults in an endemic region of Midwest Brazil. *Jornal de Pediatria*.

[20] Tarmisi A., Arifuddin A., Epidemiologi B., Kedokteran F., Tadulako U., Kunci K. (2016). High endemic risk analysis in the hot water village of Pirigi Barat district. *Parigi Moutong District*.

[21] Fernandes S. A., Bassani L., Nunes F. F., Aydos M. E. D., Alves A. V., Marroni C. A. (2012). Nutritional assessment in patients with cirrhosis. *Arquivos de Gastroenterologia*.

